# The effects of vitamin B12 supplementation on metabolic profile of patients with non-alcoholic fatty liver disease: a randomized controlled trial

**DOI:** 10.1038/s41598-022-18195-8

**Published:** 2022-08-18

**Authors:** Hamid Reza Talari, Mohamad Reza Molaqanbari, Milad Mokfi, Mohsen Taghizadeh, Fereshteh Bahmani, Seyed Mohammad Hossein Tabatabaei, Nasrin Sharifi

**Affiliations:** 1 Department of Radiology, Faculty of Medicine, Kashan University of Medical Sciences, Kashan, Iran; 2 Department of Internal Medicine, Faculty of Medicine, Kashan University of Medical Sciences, Kashan, Iran; 3 Research Center for Biochemistry and Nutrition in Metabolic Diseases, Basic Science Research Institute, Kashan University of Medical Sciences, Kashan, 87159-73474 Iran

**Keywords:** Liver diseases, Non-alcoholic fatty liver disease, Nutrition

## Abstract

The present study is the first effort to evaluate the effects of vitamin B12 supplementation on the serum level of liver enzymes, homocysteine, grade of hepatic steatosis, and metabolic profiles in patients with non-alcoholic fatty liver disease (NAFLD). Forty patients with NAFLD were enrolled in a double-blind placebo-controlled trial to receive either one oral tablet of vitamin B12 (1000 µg cyanocobalamin) or a placebo per day for 12 weeks. We investigated serum levels of homocysteine, aminotransferases, fasting blood glucose (FBG), lipids, malondialdehyde (MDA), and homeostasis model assessment of insulin resistance (HOMA-IR). The grade of liver steatosis and fibrosis was measured by real-time 2-dimensional shear wave elastography. Vitamin B12 supplementation significantly decreased serum levels of homocysteine compared to placebo (medians: − 2.1 vs. − 0.003 µmol/l; *P* = 0.038). Although serum alanine transaminase (ALT) in the vitamin B12 group decreased significantly, this change did not reach a significant level compared to the placebo group (medians: − 7.0 vs. 0.0 IU/l; *P* > 0.05). Despite the significant within-group decrease in FBG, MDA, and liver steatosis in the vitamin B12 group, between-group comparisons did not reveal any significant difference. Vitamin B12 supplementation might decrease serum levels of homocysteine in patients with NAFLD. The fasting blood glucose and serum levels of MDA were significantly improved in the trial group who received vitamin B12. However, these changes did not reach a significant level compared to the placebo group. In this respect, further studies with larger sample sizes, different doses, and types of vitamin B12 will reveal additional evidence.

**Trial Registration:** At http://irct.ir/ as IRCT20120718010333N5 on December 25, 2019.

## Introduction

Non-alcoholic fatty liver disease (NAFLD) is defined as triglyceride accumulation in more than 5% of hepatocytes in the absence of alcohol consumption^1^. This disease has a broad spectrum from simple steatosis to steatohepatitis and fibrosis^2^. Insulin resistance, obesity, decreased fatty acid oxidation, dietary factors, and environmental and bacterial pollution are known causes of NAFLD^1^.

Deficiency in dietary intakes of methyl donors such as methionine, choline, and folic acid are investigated for their possible effects on NAFLD development^3^. Methionine in the form of s-adenosyl methionine (SAMe) may have essential roles in hepatocyte metabolism^4^. In the liver, methionine is synthesized from homocysteine by the enzyme methionine synthase (MS). In this reaction, 5-methyl-tetrahydrofolate transfers its methyl group to cobalamin (vitamin B12) to form methyl-cobalamin bound to MS and activates the enzyme^4^. Then, the activated methyl group is transferred from methyl-cobalamin to homocysteine to produce methionine. In the next step, methionine is converted to SAMe by methionine adenosyltransferase^5^. The availability of SAMe in hepatocytes leads to phosphatidylcholine production^5^. Therefore, folate and cobalamin deficiency may decline hepatic levels of SAMe and phosphatidylcholine (PC)^6^. The alteration in the availability of both SAMe and PC has been shown in the etiology of fatty liver disease^3^. To our knowledge, rare human studies have evaluated the association between serum vitamin B12 and NAFLD. However, these studies have revealed inconsistent results. Some researchers have reported similar vitamin B12 levels in NAFLD patients compared to controls^7^, whereas others showed lower or even higher levels^7–9^. More human studies are needed to elucidate the relationships between folate or cobalamin deficiency and NAFLD.

Accordingly, for the first time, the present study evaluated the effects of vitamin B12 supplementation on serum liver enzymes, grade of hepatic steatosis, and metabolic profiles in patients with NAFLD in a randomized controlled trial model.

## Materials and methods

### Study participants

Men and women aged between 18 and 80 years diagnosed with NAFLD by ultrasound who had an increased serum level of alanine transaminase (ALT) were recruited from the Gastroenterology Clinic and Radiology Center of Shahid Beheshti Hospital, Kashan University of Medical Sciences. Serum ALT levels higher than 30 U/l in men and higher than 19 U/l in women were used as inclusion criteria^10^. Diabetic patients with NAFLD were included if they had been recently diagnosed with diabetes mellitus and did not use blood glucose-lowering medications. In the case of taking insulin and metformin, it should have passed at least 6 months from the start of treatment. During that period, the metformin and insulin dose must not have changed. Exclusion criteria included the following: (1) alcohol consumption greater than 20 g/day; (2) any kinds of chronic liver diseases; (3) having a history of jejunoileal bypass surgery or gastroplasty; (4) using total parenteral nutrition during the last 6 months; (5) taking hepatotoxic drugs; (6) history of having hypothyroidism, renal failure, and Cushing’s syndrome; and (7) consumption of vitamin E, vitamin B12, and folate supplements during the last 3 months. Due to not finding any study similar to ours, we used the data needed for sample size calculation from Kwok et al. study, which examined the effects of vitamin B12 supplementation on cognitive function in patients with type 2 diabetes (T2D)^11^. In the present study, 18 subjects were assigned to each of the considered two groups to detect a change of 4.7 µmol/l in serum levels of homocysteine with 90% power and 5% significance. The possible dropout was considered by adding a 10% extra subjects to the sample size. The Ethics Committee of Kashan University of Medical Sciences approved the study protocol (Registration No. IR.KAUMS.MEDNT.REC.1398.088). All participants signed the written informed consent before entering the study. The procedures used in this study adhered to the tenets of the Declaration of Helsinki. The trial was registered with the Iranian Registry of Clinical Trials on 25/12/2019 (http://www.irct.ir, registered code: IRCT20120718010333N5).

### Study design

This study was a randomized double-blind placebo-controlled trial of parallel design. Based on the blocked randomization method, the participants were randomly divided into two groups (1:1 ratio) with 4-subject randomization blocks. The sequence of blocks was generated with a computer random number generator. One researcher who was not clinically involved in the trial packaged the supplements and placebos in numbered bottles based on a random list. Another person with no role in the next steps of the trial and unaware of random sequences assigned patients to the numbered bottles of tablets. Randomization and allocation were concealed from the researchers and participants until the statistical analysis was completed. Participants received one oral tablet containing 1000 µg of cyanocobalamin (HealthAid, Co, UK) or a placebo (Barij Essence Co, Kashan, Iran) daily for 12 weeks. The placebo pill was similar in color, shape, size, and packaging to vitamin B12 and contained edible starch. Compliance was assessed by unused tablets, which were returned to the researchers. All study participants were recommended to limit high-carbohydrate and high-fat foods and increase their physical activity levels as standard treatment for NAFLD^12,13^. We called patients every 2 weeks to remind them of the supplements and record any side effects.

Height, weight, and body mass index (BMI) were measured at baseline and the end of the study. In the beginning, demographic information and records of diseases, medications, and dietary supplements were acquired from the patients. Subjects were advised no longer to take any vitamin B-complex, folate, and different dietary supplements in the course of the study and to inform researchers if there was a change in the type or dose of medication used. The dietary intake was assessed by obtaining a 2-day 24-h dietary recall from the participants before and after the study. The mean daily nutrient intake was calculated using modified Nutritionist IV software. In addition, each subject’s physical activity was assessed using the short form of the International Physical Activity Questionnaire (IPAQ) at the study’s beginning and end^14^.

### Primary outcomes

The primary outcomes included: (1) changes in the grade of liver steatosis and fibrosis; (2) changes in the serum levels of ALT and aspartate transaminase (AST); (3) changes in the serum levels of homocysteine, and (4) changes in the homeostasis model assessment of insulin resistance (HOMA-IR).

### Elastography assessments

The real-time B-mode ultrasound-based attenuation imaging (ATI) and the real-time 2-dimensional shear wave elastography (2D-SWE) were used to examine the liver steatosis and fibrosis pre- and post-study. These examinations were performed using the Aixplorer system (Supersonic Imagine, Aix en Provence, France) with a C6-1 curvilinear probe. In this regard, 2D-SWE has been developed as a non-invasive method to assess liver fibrosis and may be superior to transient elastography (TE) in detecting significant fibrosis in NAFLD^15^. In addition, real-time B-mode ultrasound (US)-based ATI, which is similar to the controlled attenuation parameter (CAP) from TE, accurately detects liver steatosis^16^. In the present study, the procedure of the 2D-SWE was similar to that of the previous research by Zaleska-Dorobisz^17^. All examinations were conducted by the same certified radiologist who was blinded to the study groups.

### Biochemical analysis

At the beginning and end of the study, 10 ml of blood sample was collected from each participant after 8–12 h of nighttime fasting. Serum ALT and AST activities were measured using the kinetic method. Also, fasting blood glucose (FBG) levels were assayed using the glucose oxidase method. Fasting insulin concentrations were assayed using the enzyme-linked immunosorbent assay (ELISA) (Monobind, USA). The HOMA-IR was calculated using the formula HOMA-IR = fasting glucose (mg/dl) × fasting insulin (µU/ml)/405. The serum homocysteine levels were determined using the ELISA method by laboratory kit (Axis-Shield Diagnostics, Scotland, UK). Serum triglyceride (TG), high-density lipoprotein-cholesterol (HDL-C), and low-density lipoprotein-cholesterol (LDL-C) concentrations were determined using enzymatic kits. Serum malondialdehyde (MDA) levels were detected by the thiobarbituric acid method^18^.

### Statistical analysis

Differences between the two groups were analyzed by chi-square (χ^2^) test for categorical variables and independent T-test or Mann–Whitney U test for continuous variables. The Paired T-test or Wilcoxon Paired Rank Test was used for within-group comparisons. The analysis of covariance (ANCOVA) test was used to determine the differences between the two groups while adjusting for baseline measurements and covariates. The analysis was performed using SPSS version 16 statistical software (SPSS Inc, Chicago, Ill). Two-sided *P* values < 0.05 were considered statistically significant.

### Ethics declarations

The Ethics Committee of Kashan University of Medical Sciences approved the study protocol (Registration No. IR.KAUMS.MEDNT.REC.1398.088). All participants signed the written informed consent to participate in the study. The trial was registered at irct.ir website (IRCT20120718010333N5).

## Results

**Figure 1:**
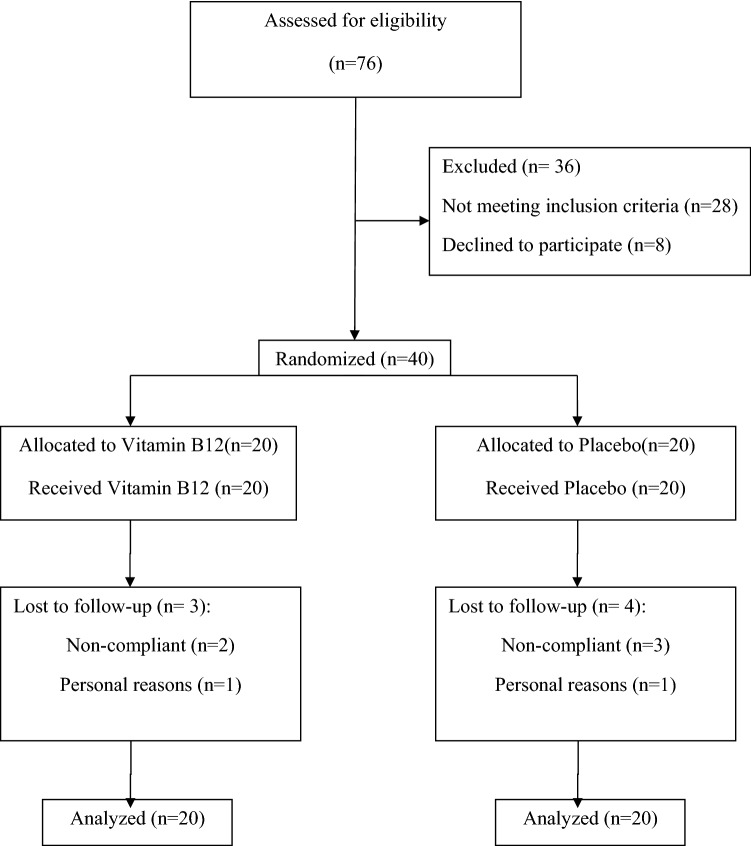
Flowchart of patients’ recruitment.

As shown in Fig. 1, some participants in each arm were withdrawn due to personal reasons or poor compliance. Therefore, 33 patients completed the study. However, all 40 participants were included in the final analysis using the intention-to-treat approach. Patients were recruited from January through February 2020. Overall, the compliance rate was high, with more than 80% of tablets taken in each trial group. The study results showed no side effects among participants who received supplements and placebos.

Baseline demographic data of the study groups are displayed in Table 1. There were no significant differences in gender, age, smoking status, history of diabetes mellitus, and use of medications between the two groups at baseline.

**Table 1:** Baseline characteristics of 40 patients with NAFLD.

Characteristics	Vitamin B12 n = 20	Placebo n = 20	*P* value
Gender (male) (%)^a^	55	60	0.75
Smoker (n)^a^	3	3	1.00
History of diabetes mellitus (%)^a^	25	5	0.18
**Use of medications [n (%)]**^**a**^			
Metformin	6 (30)	5 (25)	0.72
ACEIs	1 (5)	1 (5)	1.00
Statins	4 (20)	1 (5)	0.34
PPIs	3 (15)	2 (10)	0.62
Age^b^	41.30 ± 6.33	39.10 ± 9.96	0.41

*ACEI*s angiotensin-converting enzyme inhibitors, *NAFL*D non alcoholic fatty liver disease, *PPIs* Proton-pump inhibitors.

^a^Data is tested by chi-square test or Fisher’s exact test.

^b^Data is expressed as mean ± standard deviation and tested by two sample t-test.

Within- and between-group changes for anthropometric and some dietary variables, as well as physical activity, are provided in Table 2. The results showed no significant differences between the groups regarding weight, BMI, and physical activity at baseline and end of the study. After finishing the trial, the changes in energy intake and dietary intakes of vitamin B12 and folate were comparable between the two groups (Table 2).

**Table 2:** Within-group and between-group comparisons of the baseline, endpoint and changes values for anthropometric, physical activity, and dietary variables in vitamin B12 and placebo groups.

Variables	Vitamin B12 group^a^ (n = 20)	Placebo group^a^ (n = 20)	*P*-value (between)^b^
**Weight (kg)**			
Baseline	87.25 (78.50, 101.22)	92.70 (85.92, 105.75)	0.307
Endpoint	85.70 (79.75, 90.06)	91.61 (86.92, 103.07)	0.063
Change^c^	− 1.55 (− 3.00, 0.40)	− 0.95 (− 4.15, 2.69)	0.607^e^
*P* value (within)^d^	0.097	0.424	
**BMI (kg/m**^**2**^**)**			
Baseline	31.42 (27.77, 34.92)	30.74 (27.83, 35.66)	0.940
Endpoint	30.67 (28.08, 33.55)	32.09 (28.70, 35.44)	0.164
Change	− 0.54 (− 1.15, 0.27)	− 0.35 (− 1.45, 0.59)	0.685^e^
*P* value (within)	0.149	0.714	
**Physical activity (MET-min/week)**^**f**^			
Baseline	0.0 (0.0, 561)	0.0 (0.0, 408)	0.905^e^
Endpoint	216 (0.0, 289)	217 (0.0, 525)	0.422^e^
Change	0.0 (− 306, 234)	0.0 (− 77, 244)	0.224^e^
*P* value (within)	0.341^e^	0.233^e^	
**Energy (Kcal/day)**			
Baseline	1975.49 ± 450.27	1994.29 ± 454.19	0.904
Endpoint	1838.91 ± 352.81	1903.95 ± 341.73	0.590
Change	− 136.58 ± 677.26	− 90.34 ± 478.33	0.822
*P* value (within)	0.404	0.462	
**Cobalamin (µg/day)**			
Baseline	3.65 ± 0.70	3.24 ± 0.82	0.124
Endpoint	2.85 ± 0.96	2.92 ± 0.88	0.841
Change	− 0.80 ± 1.35	− 0.32 ± 1.09	0.270
*P* value (within)	0.022	0.253	
**Folic acid (µg/day)**			
Baseline	304.95 ± 112.11	267.53 ± 80.16	0.277
Endpoint	198.61 ± 90.41	156.84 ± 77.27	0.160
Change	− 106.33 ± 178.08	− 110.68 ± 107.52	0.931
*P* value (within)	0.021	0.001	

*BMI* body mass index, *MET* metabolic equivalent of task.

^a^Values are expressed as mean ± standard deviation or median (25th, 75th percentiles).

^b^*P*-value for comparing the values between the study groups at baseline, at the endpoint and the change from baseline. Two sample t-test and Mann–Whitney U test were used for parametric and non-parametric comparisons, respectively.

^c^Endpoint values minus the baseline ones.

^d^*P*-value for comparing baseline with the end point values within each group. Paired sample t-test and Wilcoxon Paired Rank test were used for parametric and non-parametric comparison, respectively.

^e^*P*-value obtained from a non-parametric test.

^f^Total MET-minutes/week = Walking (3.3METs × min × days) + Moderate intensity (4 METs × min × days) + Vigorous intensity(8METs*min*days), based on the short form of International Physical Activity Questionnaire (IPAQ).

Within- and between-group comparisons for the primary outcomes of the study are given in Table 3. The mean value of the steatosis score decreased significantly within both groups, although the changes were not significantly different. Besides, the fibrosis score did not change significantly according to within- and between-group analyses. Although serum levels of ALT in the vitamin B12 group decreased significantly, this change did not reach a significant level compared to the placebo group. Despite a substantial decrease in serum levels of AST in both study groups, these changes were comparable. Supplementation with vitamin B12 significantly decreased serum levels of homocysteine compared to placebo at the end of 12 weeks. This result remained significant between the study groups even after adjusting for baseline values, age, and having a history of diabetes. In the vitamin B12 group, the levels of FBG significantly decreased; however, the between-group comparison revealed a nonsignificant *P*-value (*P* = 0.167). Such a decrease was observed in serum levels of fasting insulin and HOMA-IR in the vitamin B12 group, although this change did not reach a significant level.

**Table 3:** Within-group and between-group comparisons of the baseline, endpoint and changes’ values for the study primary outcomes in vitamin B12 and placebo groups.

Variables	Vitamin B12 group^a^ (n = 20)	Control group^a^ (n = 20)	*P* value^b^ (between)	*P* value^c,d^ (ANCOVA)
**Steatosis values (dB/cm/MHz)**				
Baseline	1.59 (1.49, 2.04)	1.54 (1.49, 1.64)	0.529^g^	
Endpoint	1.30 (1.08, 1.52)	1.30 (1.12, 1.49)	0.516	0.802
Change^e^	− 0.41 (− 0.54, − 0.14)	− 0.30 (− 0.65, − 0.02)	0.417^g^	
*P* value (within)^f^	< 0.001^g^	0.001^g^		
**Fibrosis values (kPa)**				
Baseline	7.05 (5.60, 7.97)	6.20 (5.57, 7.40)	0.567	
Endpoint	6.60 (5.22, 7.40)	6.50 (5.96, 7.07)	0.968^g^	0.821
Change	− 0.35 (− 2.20, 1.70)	0.10 (− 0.95, 0.85)	0.788	
*P* value (within)	0.587^g^	0.727^g^		
**FBG (mg/dl)**				
Baseline	99.50 (91.00, 113.50)	92.50 (87.00, 97.75)	0.091^g^	
Endpoint	96.77 (89.00, 100.98)	88.50 (81.75, 99.99)	0.134^g^	0.167
Change	− 5.00 (− 15.50, 1.75)	− 1.50 (− 7.25, 2.94)	0.265^g^	
*P* value (within)	0.025^g^	0.360^g^		
**Fasting serum insulin (µU/ml)**				
Baseline	13.46 (11.47, 19.73)	15.03 (9.59, 18.48)	0.588^g^	
Endpoint	14.61 (9.28, 20.41)	12.21 (9.59, 18.39)	0.818^g^	0.740
Change	− 1.46 (− 5.48, 2.92)	− 0.21 (− 4.59, 6.84)	0.570^g^	
*P* value (within)	0.351^g^	0.970^g^		
**HOMA-IR**				
Baseline	3.74 (2.75, 6.15)	3.50 (2.06, 4.49)	0.351^g^	
Endpoint	3.94 (2.42, 6.28)	3.12 (2.11, 7.05)	0.766^g^	0.408
Change	− 0.23 (− 0.88, 2.34)	0.06 (− 1.64, 2.49)	0.534^g^	
*P* value (within)	0.794^g^	0.550^g^		
**ALT (IU/l)**				
Baseline	44 (24, 70)	33 (20, 60)	0.203^g^	
Endpoint	30 (19, 47)	30.5 (19, 40)	0.978^g^	0.824
Change	− 7 (− 17, − 0.75)	0.0 (− 11, .8)	0.559	
*P* value (within)	0.005^g^	0.102^g^		
**AST(IU/l)**				
Baseline	31 (26, 36)	27.5 (21, 40)	0.357^g^	
Endpoint	19.5 (16, 23)	21 (16, 23)	0.807^g^	0.859
Change	− 12 (− 14.7, − 4.9)	− 8 (− 8, − 3)	0.516^g^	
*P* value (within)	< 0.001^g^	< 0.001^g^		
**Serum homocysteine (µmol/l)**				
Baseline	15.1 (12, 17.8)	14.5 (13, 16.8)	0.933^g^	
Endpoint	11.5 (10, 16.7)	14.1 (12.7, 16.1)	0.258	0.038
Change	− 2.1 (− 4.3, − 0.003)	− 0.003 (− 1.7, 0.47)	0.036	
*P* value (within)	0.005	0.351^g^		

*ALT* alanin transaminase, *ANCOVA* analysis of covariance, *AST* aspartate transaminase, *FBG* fasting blood glucose; *HOMA-IR* homeostatic model assessment of insulin resistance.

^a^Values are expressed as mean ± standard or median (25th, 75th percentiles).

^b^*P*-value for comparing the values between the study groups at baseline, at the endpoint and the change from baseline. Two sample t-test and Mann–Whitney U test were used for parametric and non-parametric comparisons, respectively.

^c^*P* value for ANCOVA test to determine the significant levels of differences between the two groups post-intervention while adjusting for baseline measurements and covariates.

^d^Adjusted for baseline values of corresponding variable, age, and the baseline values of FBG.

^e^Endpoint values minus the baseline ones.

^f^*P*-value for comparing baseline with end point values within each group. Paired sample t-test and Wilcoxon Paired Rank test were used for parametric and non-parametric comparison, respectively.

^g^*P*-value obtained from a non-parametric test.

Within- and between-group comparisons for the secondary outcomes of the study are shown in Table 4. Vitamin B12 supplementation did not change fasting serum levels of TG and LDL-c. Although the serum HDL-c levels decreased near the significant levels in both trials’ arms, no significant difference was observed between the groups. The serum levels of MDA were reduced significantly in the vitamin B12 group; however, this change was not significant compared to the placebo group (Table 4).

**Table 4:** Within-group and between-group comparisons of the baseline, endpoint and changes’ values for the study secondary outcomes in vitamin B12 and placebo groups.

Variables	Vitamin B12 group^a^ (n = 20)	Control group^a^ (n = 20)	*P* value^b^ (between)	*P* value^c,d^ (ANCOVA)
**TG (mg/dl)**				
Baseline	170 (126, 256)	140 (103, 177)	0.127^g^	
Endpoint	153 (121, 186)	149 (108, 173)	0.607	0.568^f^
Change^e^	− 12 (− 45, 35)	4.5 (− 34, 21)	0.577	
*P* value (within)^f^	0.446	0.940^ g^		
**LDL-c (mg/dl)**				
Baseline	96 (80, 108)	99 (83, 115)	0.732	
Endpoint	97 (80, 115)	102 (95, 114)	0.559	0.597^f^
Change	13 (− 12, 29)	7.5 (− 10, 21)	0.854	
*P* value (within)	0.678	0.319		
**HDL-c (mg/dl)**				
Baseline	41.5 (36.2, 46)	45 (39.2, 52.7)	0.230	
Endpoint	40.9 (32.2, 46.8)	43 (40.1, 45.7)	0.471	0.783^f^
Change	− 1.9 (3.8, 1.6)	− 2 (− 8, 2)	0.635^g^	
*P* value (within)	0.054	0.045		
**MDA (ng/ml)**				
Baseline	4.37 ± 0.89	4.38 ± 1.05	0.969	
Endpoint	3.77 ± 0.56	4.17 ± 0.66	0.049	0.119
Change	− 0.60 ± 1.14	− 0.21 ± 1.13	0.292	
*P* value (within)	0.029	0.402		

*ANCOVA* analysis of covariance, *HDL-c* high density lipoprotein-cholesterol, *LDL-C* low density lipoprotein cholesterol, *MDA* Malondialdehyde, *TG* triglyceride.

^a^Values are expressed as mean ± standard deviation or median (25th, 75th percentiles).

^b^*P*-value for comparing the values between the study groups at baseline, at the endpoint and the change from baseline. Two sample t-test and Mann–Whitney U test were used for parametric and non-parametric comparisons, respectively.

^c^*P* value for ANCOVA test to determine the significant levels of differences between the two groups post-intervention while adjusting for baseline measurements and covariates.

^d^Adjusted for baseline values of corresponding variable, age, and the baseline values of FBG.

^e^Endpoint values minus the baseline ones.

^f^*P*-value for comparing baseline with end point values within each group. Paired sample t-test and Wilcoxon Paired Rank test were used for parametric and non-parametric comparison, respectively.

^g^*P*-value obtained from a non-parametric test.

## Discussion

Daily supplementation with one oral tablet consisting of 1000 µg cyanocobalamin for 12 weeks decreased the serum level of homocysteine compared to the placebo in patients with NAFLD. Some previous experimental and cross-sectional studies revealed an association between NAFLD and hyperhomocysteinemia^19–22^. In contrast, some others reported comparable serum homocysteine concentrations between patients with NAFLD and healthy controls^7,23^. Dai et al. conducted a meta-analysis to estimate whether NAFLD patients have higher serum levels of homocysteine than healthy controls^24^. They reported that patients with NAFLD had significantly higher serum homocysteine levels than healthy controls^24^. In addition, NAFLD was associated with a significantly increased risk of hyperhomocysteinemia (Odds ratio = 5.01, CI 1.69, 15.32)^24^. In this respect, higher serum homocysteine levels may alter intracellular lipid metabolism, resulting in liver steatosis^25^. In addition, accumulated homocysteine in hepatic cells causes endoplasmic reticulum stress and disturbs the sterol response pathway, leading to NAFLD progression^22^. The deficiency of some vitamins such as folate, vitamin B12, and vitamin B6 increases the serum and tissue levels of homocysteine. Folate and cobalamin, through methyl donation, convert homocysteine to methionine and decrease its serum and tissue levels^26^. The present study showed that vitamin B12 supplementation in NAFLD patients decreased serum homocysteine levels significantly compared to the placebo, even after adjusting for some covariates. Hence, it seems that vitamin B12 supplementation might have beneficial effects on NAFLD outcomes by decreasing hyperhomocysteinemia.

In the present study, the mean steatosis score decreased significantly in both trial groups after supplementation. However, these changes were not significantly different between them. This parallel decrease in steatosis score might be due to the mild weight loss of both study groups’ participants, which would partially mask the main effect of vitamin B12. Findings from previous animal and cross-sectional studies showed possible roles of vitamin B12 deficiency in hepatic steatosis. The results of an experimental study revealed that methyl donor supplementation, in which folate and vitamin B12 were among them, could improve obesogenic diet-induced hepatic triglyceride accumulation in a sample of rats^27^. A cross-sectional study that compared 45 cases of NAFLD with 30 healthy controls found lower vitamin B12 in patients with grade 2 and grade 3 of liver steatosis^8^. However, Polyzos et al. observed similar vitamin B12 levels between patients with NAFLD and healthy controls^9^.

Furthermore, vitamin B12 levels were not associated with the severity of liver disease^9^. Vitamin B12 might reduce hepatic fat accumulation through some proposed mechanisms. Methyl donor supplementation could modify DNA methylation and subsequent changes in genes responsible for reproducing enzymes involved in de novo hepatic lipogenesis, such as fatty acid synthase^28^. Besides, vitamin B12 and folate deficiency may impair phosphatidylcholine production, leading to the accumulation of hepatic TG due to a reduction in VLDL secretion^29,30^.

Although serum levels of ALT in the vitamin B12 group decreased significantly in the present study, this change did not reach a significant level compared to the placebo group. There is a lack of clinical trials investigating the effect of vitamin B12 supplementation on liver enzymes in NAFLD patients. A negative correlation was obtained between vitamin B12 levels and serum concentrations of ALT in a cross-sectional study^8^. Moreover, in an animal study, Abdulkhaleq et al. showed that pre-treatment with vitamin B12 inhibited the increase in ALT serum levels in rats in which hepatotoxicity was induced by acetaminophen^31^.

Evidence has revealed that MDA, a marker of lipid peroxidation, was increased in patients with NAFLD^32,33^. In our trial, the serum levels of MDA decreased significantly in the vitamin B12 group; however, this change was nonsignificant compared to the placebo group. This finding could be in line with a significant reduction in homocysteine levels in the present trial group who received vitamin B12. The results of previous studies have shown that therapies such as folate supplementation can decrease MDA levels by lowering the serum levels of homocysteine^34–36^. It is not known if vitamin B12 has a direct and independent beneficial effect on lipid peroxidation. Further studies with different designs could reveal more facts in this regard.

Insulin resistance is one of the crucial factors in NAFLD pathogenesis^37^. In the present study, supplementation with vitamin B12 did not significantly improve the markers of glucose metabolism. However, the decrease in serum levels of FBG was more pronounced in vitamin B12 than in the placebo group. The inverse association between serum levels of vitamin B12 and insulin resistance (IR) has been reported by some researchers^38–40^. In two previous clinical trials, the combination use of vitamin B12 (500 µg/day) and folic acid (5 mg/day) for about eight weeks led to a significant improvement in FBG levels and HOMA-IR^41,42^. However, the independent effects of vitamin B12 on IR could not be interpreted from the mentioned trials due to combination therapy. In a recent multi-arm trial by Satapathy et al., participants with T2D in one arm of the trial (n = 20) received 500 mcg/day of methylcobalamin for eight weeks^43^. At the end of the study, the results showed significant improvements in plasma insulin and insulin resistance in this group compared to the placebo arm^43^. Using different types of supplements (methylcobalamin vs. cyanocobalamin) and different study populations would be the reasons for finding different results between our study and Satapathy et al.^43^. The proposed mechanisms for the beneficial effects of vitamin B12 and folic acid on insulin resistance are related to the mediating role of homocysteine^42,44^. Homocysteine might alter insulin signaling pathways as it could inhibit the tyrosine phosphorylation of insulin receptor and its substrate insulin receptor substrate − 1^44^.

The strength of the present study is that it is the first double-blind randomized controlled trial that has evaluated the effects of vitamin B12 on NAFLD. We adjusted confounding variables such as daily energy, vitamin B12, folate intake, and physical activity. In addition, the observed significant decrease in serum levels of homocysteine showed acceptable compliance with supplementation in our participants.

Although liver biopsy precisely detects the histological changes in the liver, we did not use this method in our research because of ethical issues. However, we used 2D-SWE as a technique that evaluates the degree of liver steatosis and fibrosis more accurately and quantitatively than ultrasonography^16^.

## Conclusions

The current study showed that supplementation with vitamin B12 (1000 µg cyanocobalamin per day) for 12 weeks among patients with NAFLD improved serum levels of homocysteine. Also, the fasting blood glucose and serum levels of MDA were significantly improved in the trial group who received vitamin B12. However, these changes did not reach a significant level compared to the placebo group. It is suggested to conduct further studies with larger sample sizes, different doses and types of vitamin B12, and different primary outcomes.

## Data Availability

The datasets generated and/or analysed during the current study are not publicly available as per the rules and regulations of the Research Center for Biochemistry and Nutrition in Metabolic Diseases at the Kashan University of Medical Science but are available upon reasonable request from the corresponding author.
